# Evaluating the impact of an evidence-based standardized nursing protocol on lower-extremity deep vein thrombosis prevention in acute stroke patients: A retrospective analysis

**DOI:** 10.1097/MD.0000000000043155

**Published:** 2025-08-01

**Authors:** Xuemei Chen, Ling Feng, Rui Wang, Shuangyan Tu, Dan Wei, Chao Peng

**Affiliations:** a Department of Neurology, West China Hospital of Sichuan University, Chengdu, Sichuan Province, China.

**Keywords:** acute stroke, clinical effectiveness, evidence-based care, lower limb deep vein thrombosis, standardized care

## Abstract

Acute stroke remains a major global health concern, contributing significantly to mortality and long-term disability. Due to factors such as immobility, endothelial injury, and hypercoagulability, these patients face an elevated risk of developing lower-extremity deep vein thrombosis (DVT). This retrospective study aimed to evaluate the efficacy of an evidence-based standardized nursing protocol in preventing DVT among acute stroke patients, comparing its outcomes with traditional nursing approaches. We analyzed data from 176 acute stroke patients admitted to our hospital between January 2022 and December 2023. The cohort was divided into 2 groups: the intervention group (n = 84), which received structured evidence-based care involving risk stratification, pharmacological and mechanical prophylaxis, early mobilization, patient education, and close monitoring, and the control group (n = 92), which received conventional nursing care. Over a 6-month follow-up period, the incidence of DVT was markedly lower in the intervention group (2.38% vs 14.13%, *P* = .005). Additionally, the intervention group demonstrated significantly fewer complications, including urinary tract infections, pneumonia, recurrent stroke, and pressure ulcers (*P* < .05). Further, patients in the intervention group exhibited superior outcomes in neurological recovery, cognitive improvement, quality of life, and mitigation of anxiety and depressive symptoms (*P* < .05). These findings underscore that an evidence-based standardized nursing protocol not only reduces DVT risk but also enhances overall recovery and well-being in acute stroke patients. The study advocates for the wider implementation of such protocols in clinical practice to optimize patient outcomes poststroke.

## 1. Introduction

Acute stroke is a major cause of mortality and long-term disability globally. According to the World Health Organization, stroke claims millions of lives annually and results in substantial long-term disability for many individuals.^[1,2]^ The World Health Organization further estimates that stroke is a leading cause of death and disability worldwide.^[3,4]^ Patients with acute stroke have an elevated risk of developing lower extremity deep vein thrombosis (DVT) due to limited mobility, endothelial injury, and hypercoagulability (often referred to as Virchow triad).^[5,6]^ The occurrence of DVT in these patients can lead to severe complications, such as pulmonary embolism, and contributes to increased morbidity, extended hospital stays, and elevated healthcare costs. Such consequences not only exacerbate the physical burden on patients but also place significant pressure on healthcare systems.^[7,8]^

Currently, strategies to prevent DVT fall into 2 main categories: pharmacological and non-pharmacological interventions.^[9]^ Pharmacological approaches, including the use of anticoagulants such as low molecular weight heparin and unfractionated heparin, have demonstrated effectiveness in reducing thrombosis risk. Non-pharmacological measures, on the other hand, involve mechanical prophylaxis, such as graduated compression stockings and intermittent pneumatic compression devices, as well as the encouragement of early mobilization.^[10]^ While these interventions theoretically have the potential to significantly reduce DVT incidence, their effectiveness in clinical practice has been suboptimal, primarily due to inconsistent implementation and mixed outcomes of nursing care measures.^[11]^

In recent years, a standardized care model grounded in evidence-based practices has gained increasing attention. This model aims to enhance the consistency and effectiveness of DVT prophylaxis through a structured and systematic care process.^[12]^ The standardized care approach includes comprehensive risk assessment, consistent use of pharmacological and mechanical prophylactic measures, initiation of early mobilization, as well as education and training for both patients and caregivers, alongside rigorous monitoring and documentation.^[13]^ This holistic model of care is expected to play a significant role in DVT prevention, ultimately enhancing the quality of care and improving clinical outcomes for acute stroke patients.

Although several studies have demonstrated the potential benefits of standardized care models for DVT prevention, most of these investigations have used prospective designs, which can be limited by time and cost constraints.^[14]^ Retrospective cohort studies, on the other hand, offer an efficient and cost-effective approach to evaluating the effectiveness of standardized care models by utilizing existing medical records and data in real clinical settings.^[15]^ Therefore, this study aims to systematically assess the effectiveness of an evidence-based standardized care model in preventing lower extremity DVT and improving other related clinical outcomes in patients with acute stroke through a retrospective cohort design. The results will provide an evidence-based foundation for clinical practice, facilitate the optimization and implementation of DVT prevention strategies, and ultimately enhance clinical outcomes and quality of life for patients with acute stroke.

## 2. Methodology

### 2.1. Study design and study population

This study was approved by the Ethics Committee of West China Hospital of Sichuan University. This retrospective cohort study aimed to evaluate the effectiveness of a standardized care model based on evidence-based practices in preventing lower limb DVT and other clinical outcomes in acute stroke patients. The study was retrospective in nature and did not involve any patient interventions. All data were anonymized and de-identified to ensure privacy and confidentiality, and were used solely for the academic publication purposes of this research. This study meets the criteria for an internationally recognized ethical exemption: data were obtained from existing clinical records, did not involve identifiable patient information, and posed no direct or potential risk to patient rights.^[16]^

Data were collected from patients treated for acute stroke in our hospital between January 2022 and December 2023. Participants were categorized into an Observation group, which received standardized evidence-based care, and a Control group, which received traditional care. A total of 84 patients were included in the Observation group and 92 in the Control group.

*Inclusion criteria*: Patients who met the diagnostic criteria for acute stroke (ischemic or hemorrhagic) as defined by the Cerebrovascular Disease Group of the Chinese Medical Association Neurology Branch. Patients received either standardized care based on evidence-based practices or traditional care during hospitalization. Age ≥ 18 years. Hospital stay of at least 3 days. Complete medical records and a minimum of 6 months post-discharge follow-up, including DVT diagnosis and relevant clinical data.

*Exclusion criteria*: Patients with a prior history of DVT or pulmonary embolism. Patients with severe hepatic or renal insufficiency (e.g., Child-Pugh class C, estimated glomerular filtration rate < 30 mL/min/1.73 m²). Patients who underwent lower limb surgery or experienced major trauma within 30 days before stroke onset. Patients with active malignancy. Patients with incomplete medical records or missing essential DVT diagnostic information. Patients transferred to the hospital <7 days after illness onset, lacking comprehensive care and follow-up data.

### 2.2. Data collection

The primary data sources included electronic medical records, which provided demographic, clinical, and outcome information; nursing records, which were reviewed to assess prophylaxis measures and interventions; imaging reports, which were used for DVT diagnosis through lower limb ultrasound; and medication records, which detailed the use of anticoagulants and other relevant treatments.

### 2.3. Modes of care

#### 2.3.1. Observation group

(1) *Risk assessment*: a comprehensive risk evaluation is conducted upon admission and repeated daily using the Caprini risk assessment model or other validated tools. (2) *Pharmacological prophylaxis*: administration of low molecular weight heparin (e.g., enoxaparin) or unfractionated heparin, with dosage adjusted according to renal function and bleeding risk, based on risk stratification and the absence of contraindications. (3) *Mechanical prophylaxis*: application of graded compression stockings and intermittent pneumatic compression devices, ensuring proper fitting and consistent use per manufacturer guidelines, with regular checks for device function and patient comfort. (4) *Early mobilization*: initiation of physiotherapy and mobilization within 24 to 48 hours of stroke onset, using an individualized plan that includes passive and active joint mobilization, sitting, and gradual walking, with daily monitoring of activity levels and progress. (5) *Education and training*: comprehensive training on DVT prevention protocols for caregivers and education for patients and families regarding the importance of activity, recognition of DVT symptoms, and preventive strategies. (6) *Monitoring and documentation*: regular follow-up on the implementation of preventive measures, patient activities, and signs of DVT, with detailed records of all interventions, patient responses, and any adverse events. (7) *Discharge rehabilitation and follow-up plan*: Development of a rehabilitation care plan for post-discharge, with monthly home visits during the first 3 months, 1 to 2 home visits between 3 to 6 months, and biweekly follow-up calls to monitor progress.

#### 2.3.2. Control group

During hospitalization, the nurse provided health education and early rehabilitation guidance to patients and established a WeChat group for ongoing support. Routine discharge instructions were provided, covering key aspects and precautions for home-based rehabilitation exercises, along with guidance on medication, diet, rest, and follow-up care. Telephone follow-ups were conducted at the 3rd and 6th months post-discharge. During each follow-up, stroke recovery was assessed, health concerns were addressed, guidance was given to patients and families, and patients were encouraged to adhere to home rehabilitation exercises, take medications as prescribed, and attend follow-up appointments.

### 2.4. Outcome indicators

*Primary outcome measure*: incidence of lower extremity DVT, diagnosed using Doppler ultrasound during hospitalization and within 60 days post-discharge. *Secondary outcome measures*: (1) occurrence of other clinical complications, including pulmonary embolism, urinary tract infections, pneumonia, recurrent stroke, pressure ulcers, and bleeding events. (2) Neurological function assessed using the National Institutes of Health Stroke Scale (NIHSS). (3) Cognitive impairment evaluated with the Montreal Cognitive Assessment (MoCA). (4) Quality of life assessed with the Stroke-Specific Quality of Life Scale (SS-QOL). (5) Depression and anxiety levels assessed using the Hospital Anxiety and Depression Scale (HADS).

### 2.5. Statistical analyses

All data analyses were conducted using SPSS version 25.0. Descriptive statistics were used to summarize demographic and clinical characteristics, presented as mean ± standard deviation (X ± SD) for continuous variables, or as frequency (n) and percentage (%) for categorical variables. Fisher exact test was used for categorical variables when the expected frequency was < 5, while the chi-square test was applied for expected frequencies ≥ 5. For continuous variables, either the *t* test or Mann–Whitney *U* test was used, depending on the distribution of the data. Statistical significance was set at *P* < .05 (*P < .05, **P < .01).

In addition to these basic analyses, multivariable regression analysis was conducted to adjust for potential confounding variables that might affect the primary and secondary outcomes. This approach allowed us to control for differences in baseline characteristics (such as age, gender, stroke type, comorbidities, etc) between the 2 groups, ensuring that the observed effects were primarily attributable to the intervention itself. For continuous variables, we employed multiple linear regression to assess the influence of standardized nursing care on outcomes such as neurological function, cognitive improvement, and quality of life. For categorical variables (e.g., incidence of DVT or complications), logistic regression was used to adjust for confounders.

## 3. Results

### 3.1. Baseline characteristics

A total of 176 patients with acute stroke were included in this study, with 84 patients in the observation group and 92 in the control group. Table 1 presents the baseline demographic and clinical characteristics of both groups. There were no significant differences between the observation and control groups in terms of age (60.78 ± 8.56 years vs 59.27 ± 10.33 years, *P* = .292), gender distribution (60.71% vs 63.04% male, *P* = .685), body mass index (BMI: 24.56 ± 3.81 kg/m² vs 24.72 ± 6.08 kg/m², *P* = .833), marital status (married 88.10% vs 86.96%, *P* = .849), education level (junior high school and below 61.90% vs 65.22%, *P* = .641), time from onset to admission (3.24 ± 0.67 hours vs 3.18 ± 0.91 hours, *P* = .617), systolic blood pressure (152.78 ± 15.99 mm Hg vs 149.33 ± 20.11 mm Hg, *P* = .208), diastolic blood pressure (88.22 ± 9.92 mm Hg vs 86.25 ± 12.01 mm Hg, *P* = .236), platelet count (171.71 ± 50.62 × 10⁹/L vs 168.09 ± 70.21 × 10⁹/L, *P* = .693), blood glucose level (6.82 ± 1.89 mM vs 7.18 ± 2.79 mM, *P* = .314), stroke type (ischemic stroke 85.71% vs 82.61%, *P* = .585), and past medical history, including hypertension (40.48% vs 44.57%, *P* = .585), smoking (55.95% vs 54.35%, *P* = .840), dyslipidemia (36.90% vs 33.70%, *P* = .668), alcohol use (38.10% vs 40.22%, *P* = .752), type 2 diabetes (28.57% vs 25.00%, *P* = .582), and coronary artery disease (29.76% vs 26.09%, *P* = .587). There were no statistically significant differences between the 2 groups, indicating good comparability in baseline characteristics (Table 1).

**Table 1:** Baseline data of the acute stroke patients (X ± SD, n/%).

Item	Observation group (n = 84)	Control group (n = 92)	*t*/*x*²	*P* value
Age (years)	60.78 ± 8.56	59.27 ± 10.33	1.058	.292
Gender			0.164	.685
Male	51 (60.71%)	58 (63.04%)		
Female	33 (39.29%)	34 (36.96%)		
BMI (kg/m²)	24.56 ± 3.81	24.72 ± 6.08	0.211	.833
Marital status			0.036	.849
Married	74 (88.10%)	80 (86.96%)		
Others	10 (11.90%)	12 (13.04%)		
Cultural levels			0.218	.641
Junior school or below	52 (61.90%)	60 (65.22%)		
High school or above	32 (38.10%)	32 (34.78%)		
Time from onset to admission (h)	3.24 ± 0.67	3.18 ± 0.91	0.501	.617
Systolic blood pressure (mm Hg)	152.78 ± 15.99	149.33 ± 20.11	1.264	.208
Diastolic blood pressure (mm Hg)	88.22 ± 9.92	86.25 ± 12.01	1.190	.236
Blood platelet count (10^9^/L)	171.71 ± 50.62	168.09 ± 70.21	0.395	.693
Glucose (mM)	6.82 ± 1.89	7.18 ± 2.79	1.010	.314
Stroke type			0.298	.585
Ischemic stroke	72 (85.71%)	76 (82.61%)		
Hemorrhagic stroke	12 (14.29%)	16 (17.39%)		
Medical history				
Hypertension	34 (40.48%)	41 (44.57%)	0.298	.585
Smoker	47 (55.95%)	50 (54.35%)	0.041	.840
Dyslipidaemia	31 (36.90%)	31 (33.70%)	0.184	.668
Alcohol use	32 (38.10%)	37 (40.22%)	0.1	.752
Type 2 diabetes mellitus	24 (28.57%)	23 (25.00%)	0.303	.582
Coronary artery disease	25 (29.76%)	24 (26.09%)	0.295	.587

### 3.2. Incidence of lower extremity DVT

During the 6-month follow-up, the incidence of lower extremity DVT was significantly lower in the observation group at 2.38% (2/84) compared to 14.13% (13/92) in the control group (*χ*²=7.78, *P* = .005). These findings indicate that a standardized nursing model based on evidence-based practices has notable effectiveness in preventing lower limb DVT in patients with acute stroke (Table 2).

**Table 2:** Incidence of lower extremity deep vein thrombosis and other complications during the 6-month follow-up period (n/%).

Item	Observation group (n = 84)	Control group (n = 92)	*x*²	*P* value
Lower extremity deep vein thrombosis	2 (2.38%)	13 (14.13%)	7.78	.005*
Pulmonary embolism	0 (0%)	2 (2.17%)	–	.498
Urinary tract infection	5 (5.95%)	16 (17.39%)	5.42	.020*
Pneumonia	4 (4.76%)	13 (14.13%)	4.39	.036*
Recurrent stroke	3 (3.57%)	11 (11.96%)	4.20	.040*
Pressure ulcer	3 (3.57%)	13 (14.13%)	5.92	.015*
Bleeding event				
Intracranial hemorrhage	1 (1.19%)	2 (2.17%)	–	1.000
Gastrointestinal bleeding	1 (1.19%)	5 (5.43%)	–	.223
Gingival bleeding	2 (2.38%)	3 (3.26%)	–	1.000
Total occurrence	14 (16.67%)	29 (31.52%)	5.21	.022*

“–” indicates Fisher exact test.

* Indicates that there is statistical significance after correction.

### 3.3. Complications

The observation group had a significantly lower incidence of multiple complications compared to the control group. Specifically, the incidence of urinary tract infection was 5.95% (5/84) in the observation group versus 17.39% (16/92) in the control group, which was statistically significant (*χ*²=5.42, *P* = .020). The incidence of pneumonia was 4.76% (4/84) compared to 14.13% (13/92) in the control group (*P* = .036). Recurrent stroke occurred in 3.57% (3/84) of the observation group versus 11.96% (11/92) in the control group (*P* = .040). The incidence of pressure ulcers was 3.57% (3/84) compared to 14.13% (13/92) in the control group (*P* = .015). The total complication rate was 16.67% (14/84) in the observation group versus 31.52% (29/92) in the control group, showing a significant difference (*χ*²=5.21, *P* = .022). These results further support the effectiveness of the standardized care model in reducing the incidence of multiple complications (Table 2).

### 3.4. Neurological function assessment

**Figure 1.**
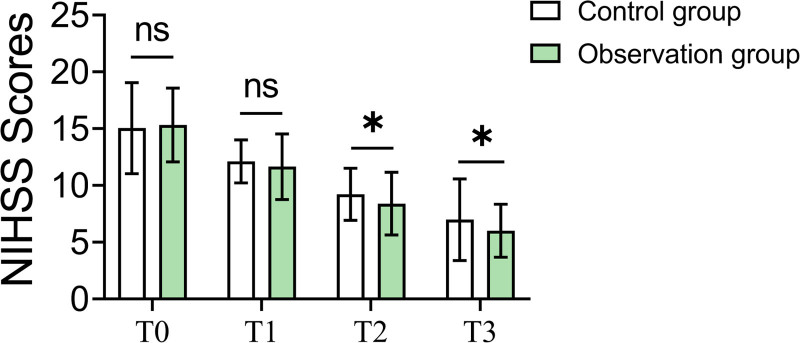
Comparison of NIHSS scores at different time points between groups. NIHSS = National Institutes of Health Stroke Scale.

The observation group demonstrated superior neurological recovery. At the 30th day (T2) and 60th day (T3) of follow-up, NIHSS scores in the observation group were 8.39 ± 2.76 and 6.01 ± 2.34, respectively, significantly lower than those in the control group, which were 9.21 ± 2.29 (*P* = .034) and 6.98 ± 3.59 (*P* = .034). These findings indicate more pronounced neurological recovery in the observation group (Table 3 and Fig. 1).

**Table 3:** NIHSS scores (X ± SD).

	Observation group (n = 84)	Control group (n = 92)	*t*	*P* value
T0	15.32 ± 3.25	15.05 ± 4.01	0.492	.623
T1	11.65 ± 2.89	12.11 ± 1.89	‐1.236	.218
T2	8.39 ± 2.76	9.21 ± 2.29	‐2.134	.034*
T3	6.01 ± 2.34	6.98 ± 3.59	‐2.139	.034*

* Indicates that there is statistical significance after correction.

### 3.5. Cognitive function assessment

**Figure 2.**
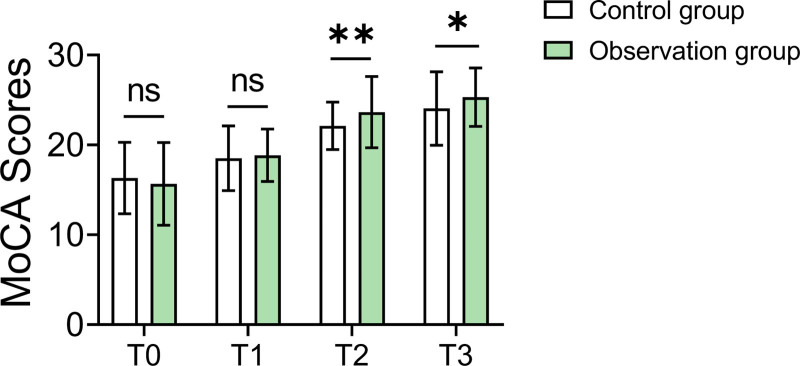
Comparison of MoCA scores at different time points between groups. MoCA = Montreal Cognitive Assessment.

The observation group showed significant improvements in cognitive function. At the 30th day (T2) and 60th day (T3) of follow-up, MoCA scores in the observation group were 23.65 ± 3.96 and 25.31 ± 3.25, respectively, significantly higher than those in the control group, which scored 22.12 ± 2.63 (*P* = .003) and 24.05 ± 4.08 (*P* = .024). These results indicate substantial improvement in cognitive function in the observation group (Table 4 and Fig. 2).

**Table 4:** MoCA scores (X ± SD).

	Observation group (n = 84)	Control group (n = 92)	t	*P* value
T0	15.66 ± 4.61	16.31 ± 3.98	‐0.996	.320
T1	18.84 ± 2.91	18.51 ± 3.61	0.670	.503
T2	23.65 ± 3.96	22.12 ± 2.63	2.990	.003*
T3	25.31 ± 3.25	24.05 ± 4.08	2.276	.024*

T0 indicates the time of admission, T1 indicates before discharge, T2 indicates 30 days after discharge, and T3 indicates 60 days after discharge.

MoCA = Montreal Cognitive Assessment.

* Indicates that there is statistical significance after correction.

### 3.6. Quality of life assessment

**Figure 3.**
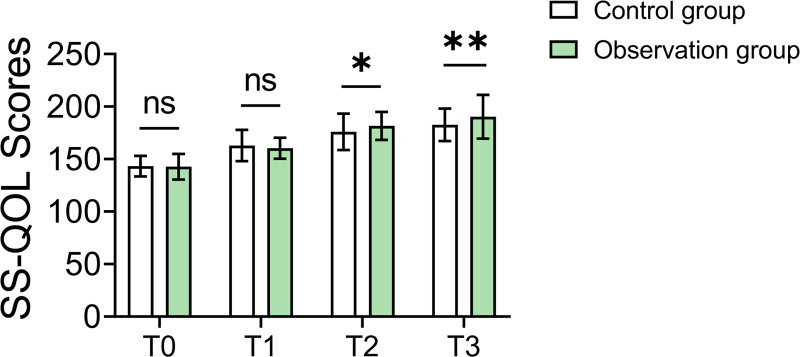
Comparison of SS-QOL scores at different time points between groups. SS-QOL = Stroke-Specific Quality of Life Scale.

The observation group demonstrated significantly better quality of life outcomes. SS-QOL scores for the observation group at the 30th day (T2) and 60th day (T3) of follow-up were 181.59 ± 13.28 and 190.25 ± 20.81, respectively, compared to the control group’s scores of 175.96 ± 17.25 (*P* = .016) and 182.65 ± 15.44 (*P* = .007). These findings indicate that the standardized care model significantly improved the quality of life for patients (Table 5 and Fig. 3).

**Table 5:** SS-QOL scores (X ± SD).

	Observation group (n = 84)	Control group (n = 92)	*t*	*P* value
T0	142.75 ± 12.21	143.26 ± 9.78	‐0.304	.762
T1	160.28 ± 9.99	162.85 ± 14.88	‐1.355	.177
T2	181.59 ± 13.28	175.96 ± 17.25	2.436	.016*
T3	190.25 ± 20.81	182.65 ± 15.44	2.730	.007*

T0 indicates the time of admission, T1 indicates before discharge, T2 indicates 30 days after discharge, and T3 indicates 60 days after discharge.

SS-QOL = Stroke-Specific Quality of Life Scale.

* Indicates that there is statistical significance after correction.

### 3.7. Anxiety and depression status assessment

**Figure 4.**
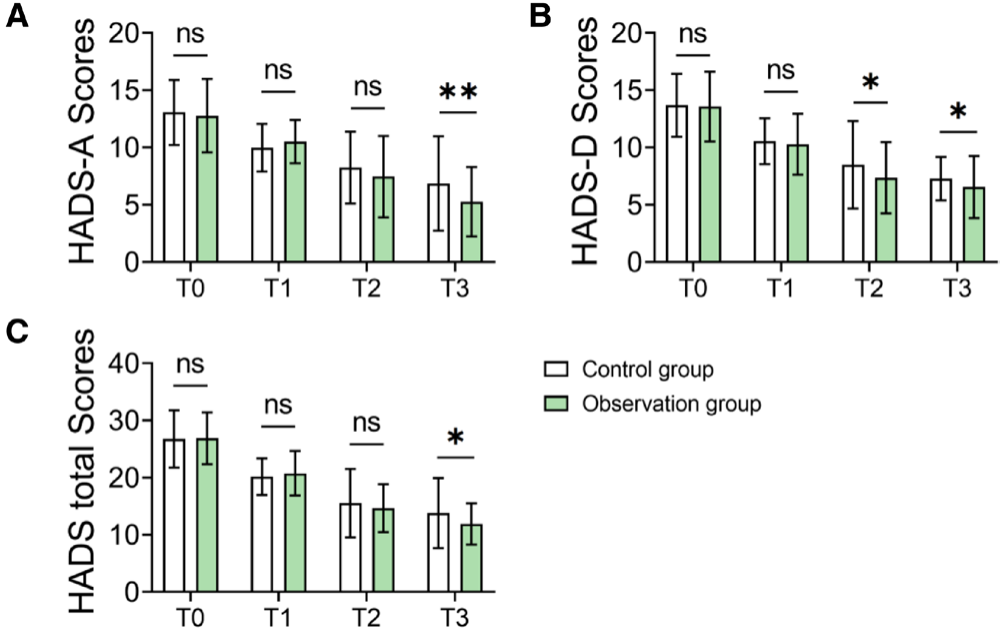
Comparison of HADS scores at different time points between groups. (A) HADS-A scores. (B) HADS-D scores. (C) HADS total scores. HADS = Hospital Anxiety and Depression Scale.

The observation group showed significant improvements in anxiety and depression scores. On the 60th day of follow-up (T3), the HADS-A score in the observation group was 5.26 ± 3.04, significantly lower than the control group’s score of 6.86 ± 4.13 (*P* = .004). Similarly, the HADS-D score in the observation group was 6.55 ± 2.71, which was significantly lower compared to 7.28 ± 1.89 in the control group (*P* = .042). These results indicate a notable reduction in anxiety and depression levels among patients who received standardized care (Tables 6–8 and Fig. 4).

**Table 6:** HADS-A scores (X ± SD).

	Observation group (n = 84)	Control group (n = 92)	*t*	*P* value
T0	12.78 ± 3.21	13.07 ± 2.84	‐0.632	.528
T1	10.52 ± 1.89	9.98 ± 2.08	1.805	.073
T2	7.45 ± 3.56	8.25 ± 3.12	‐1.580	.116
T3	5.26 ± 3.04	6.86 ± 4.13	‐2.944	.004*

HADS = Hospital Anxiety and Depression Scale.

* Indicates that there is statistical significance after correction.

**Table 7:** HADS-D scores (X ± SD).

	Observation group (n = 84)	Control group (n = 92)	*t*	*P* value
T0	13.58 ± 3.05	13.69 ± 2.75	‐0.250	.803
T1	10.29 ± 2.66	10.55 ± 1.99	‐0.729	.467
T2	7.36 ± 3.11	8.49 ± 3.81	‐2.163	.032*
T3	6.55 ± 2.71	7.28 ± 1.89	‐2.055	.042*

HADS = Hospital Anxiety and Depression Scale.

* Indicates that there is statistical significance after correction.

**Table 8:** HADS total scores (X ± SD).

	Observation group (n = 84)	Control group (n = 92)	*t*	*P* value
T0	26.88 ± 4.52	26.76 ± 5.02	0.167	.867
T1	20.76 ± 3.89	20.18 ± 3.21	1.073	.285
T2	14.66 ± 4.20	15.55 ± 5.99	‐1.149	.252
T3	11.90 ± 3.63	13.82 ± 6.13	‐2.551	.012*

T0 indicates the time of admission, T1 indicates before discharge, T2 indicates 30 days after discharge, and T3 indicates 60 days after discharge.

HADS = Hospital Anxiety and Depression Scale.

* Indicates that there is statistical significance after correction.

## 4. Discussion

The study results indicated that the incidence of lower limb DVT was significantly lower in the observation group (2.38%) compared to the control group (14.13%, *P* = .005). Additionally, the observation group had significantly lower rates of complications, including urinary tract infections, pneumonia, recurrent stroke, and pressure ulcers (*P* < .05). Regarding neurological recovery (NIHSS score), cognitive function (MoCA score), quality of life (SS-QOL score), and anxiety and depression levels (HADS score), the observation group demonstrated significantly greater improvements compared to the control group, particularly at the 30th and 60th days of follow-up, with all differences reaching statistical significance.

The results of this study align with existing literature and support the effectiveness of standardized care models based on evidence-based concepts in preventing DVT.^[17]^ Numerous prospective studies and randomized controlled trials (RCTs) have demonstrated that a systematic care process can significantly reduce DVT incidence. For instance, Muñoz et al reported a significant reduction in DVT incidence among stroke patients following the implementation of a standardized care process in a prospective study.^[18]^ Similarly, a study by Liu et al indicated that standardized care models not only effectively prevent DVT but also improve overall clinical outcomes, including reducing infection rates and pressure ulcer incidence.^[19]^ However, some studies have highlighted that the effectiveness of care interventions may be influenced by factors such as nursing staff training, resource allocation, and patient compliance.^[20]^ This study further validated the effectiveness of the standardized care model in a real clinical setting using a retrospective cohort design, thereby enhancing its external validity.

The standardized care model has effectively reduced the incidence of DVT and its related complications by implementing evidence-based measures, including comprehensive risk assessment, standardized pharmacological and mechanical prophylaxis, early mobilization, education and training for caregivers and patients, and follow-up visits. The success of this model can be attributed to the following key aspects: (1) *comprehensive risk assessment*: tools such as the Caprini Risk Assessment Model enable accurate identification of high-risk patients, ensuring the relevance and efficacy of preventive interventions. (2) *Standardized prophylaxis*: consistent pharmacological and mechanical prophylaxis protocols reduce variability in care, enhancing the overall effectiveness of DVT prevention. (3) *Early activity promotion*: timely initiation of physiotherapy and mobilization improves circulation and reduces thrombosis risk. (4) *Education and training*: comprehensive education for nursing staff, patients, and their families has increased adherence to preventive measures and enhanced patient compliance. (5) *Monitoring and record-keeping*: regular tracking of preventive measures and patient health, along with prompt identification and management of potential issues, has ensured ongoing improvements in care quality.

This study, based on real-world medical records, demonstrates the practical effectiveness of a standardized care model, providing high external validity. The inclusion of 176 acute stroke patients enhances the reliability of the findings. In addition to assessing DVT incidence, multiple clinical outcome indicators were evaluated, offering a comprehensive view of the overall impact of the care model.

However, as a retrospective cohort study, there is potential for selection and information bias, which may affect the internal validity of the findings.^[21]^ The completeness and accuracy of data are dependent on existing medical records, which may contain missing or insufficiently documented information.^[22]^ Additionally, this is a single-center study, which may limit the generalizability of the results to other healthcare settings, such as primary hospitals or institutions in different regions. The clinical applicability of our findings could be influenced by variations in healthcare systems, nursing practices, and patient populations across different settings. Therefore, future studies across multiple centers with more diverse populations are necessary to enhance the external validity of these results.

While our study supports the effectiveness of the standardized care model, the lack of randomization is a limitation. Further RCTs are recommended to validate the results. RCTs, with their ability to minimize selection biases, would provide more robust evidence of the model’s impact on DVT prevention and other clinical outcomes. These studies could also explore the cost-effectiveness and long-term sustainability of such a standardized care approach in diverse clinical environments.

This retrospective cohort study demonstrated that a standardized care model based on evidence-based concepts was significantly more effective than the traditional care model in preventing lower limb DVT and improving clinical outcomes in acute stroke patients. The standardized care model not only reduced the incidence of DVT and related complications but also facilitated neurological and cognitive recovery, improved patient quality of life, and alleviated anxiety and depression through a systematic approach. These findings suggest that the standardized care model has significant clinical application value and merits broader implementation across healthcare settings. Future studies should further validate its effectiveness, optimize nursing processes, and explore additional strategies to support patients’ full recovery.

## Author contributions

**Conceptualization:** Xuemei Chen, Chao Peng.

**Data curation:** Ling Feng, Chao Peng.

**Formal analysis:** Rui Wang, Chao Peng.

**Investigation:** Shuangyan Tu, Chao Peng.

**Methodology:** Dan Wei, Chao Peng.

**Supervision:** Xuemei Chen, Chao Peng.

**Writing – original draft:** Xuemei Chen, Chao Peng.

**Writing – review & editing:** Xuemei Chen, Chao Peng.
